# Supplementary material for: Categorizing Acute Respiratory Distress Syndrome with Different Severities by Oxygen Saturation Index
Source: Diagnostics (Basel). 2023 Dec 24;14(1):37. doi: 10.3390/diagnostics14010037 (PMC10795683; doi:10.3390/diagnostics14010037)
Supplement: Supplementary file 1 [file diagnostics-14-00037-s001.zip › Table s1 Mortality of various category of ARDS patient defined by Berlin definition, OI and OSI.docx]

**Table s1 Mortality of various category of ARDS patient defined by Berlin definition, OI and OSI**

|  | Mortality at 28-day | Mortality at 90-day |
| --- | --- | --- |
| Berlin definition |  |  |
| Mild | 14 (60.9%) | 17 (73.9%) |
| Moderate | 58 (40.0%) | 73 (50.3%) |
| Severe | 102 (54.0%) | 127 (67.2%) |
| OI |  |  |
| Mild | 57 (39.6%) | 73 (50.7%) |
| Moderate | 45 (42.1%) | 62 (57.9%) |
| Severe | 72 (67.3%) | 82 (76.6%) |
| OSI |  |  |
| Mild | 63 (41.2%) | 83 (54.2%) |
| Moderate | 47 (41.2%) | 66 (57.9%) |
| Severe | 76 (67.9%) | 84 (75.0%) |

Values are expressed as number (%)
