# Supplementary material for: Categorizing Acute Respiratory Distress Syndrome with Different Severities by Oxygen Saturation Index
Source: Diagnostics (Basel). 2023 Dec 24;14(1):37. doi: 10.3390/diagnostics14010037 (PMC10795683; doi:10.3390/diagnostics14010037)
Supplement: Supplementary file 1 [file diagnostics-14-00037-s001.zip › Table s2 Univariate analysis of factors associated with 90 day mortality.docx]

**Table s2** Univariate analysis of selected variables potentially associated with 90-day mortality

|  | Survival | Death | HR (95% CI) | P-value |
| --- | --- | --- | --- | --- |
| Age (year), mean ± SD | 60 ±17 | 67 ±15 | 1.01 (1.00-1.02) | 0.04* |
| Male, No. (%) | 92 (63) | 170 (73) | 1.42 (1.06-1.89) | 0.02* |
| BMI, median (IQR), (Kg/m^2^) | 23 (21-27) | 23 (20-26) | 0.97 (0.94-1.00) | 0.03* |
| APACHE II Score, median (IQR) | 23 (17-30) | 25 (19-29) | 1.00 (0.99-1.02) | 0.61 |
| SOFA score, median (IQR) | 7 (5-10) | 7 (5-10) | 1.02 (0.99-1.06) | 0.21 |
| Lung injury score, median (IQR) | 11 (10-12) | 11 (10-12) | 0.99 (0.93-1.06) | 0.78 |
| Severity by Berlin definition |  |  |  |  |
| Mild, No. (%) | 6 (4) | 17 (8) | 1 |  |
| Moderate, No. (%) | 72 (51) | 73 (34) | 0.54 (0.32-0.92) | 0.02* |
| Severe, No. (%) | 62 (44) | 127 (59) | 0.89 (0.53-1.47) | 0.64 |
| OI, median (IQR) | 18 (13-26) | 23 (16-34) | 1.03 (1.02-1.04) | <0.01* |
| OI < 17.8 (mild), No. (%) | 71 (50) | 73 (34) | 1 |  |
| 17.8 < OI < 28.8 (moderate), No. (%) | 45 (32) | 62 (29) | 1.23 (0.87-1.72) | 0.24 |
| OI > 28.8 (severe), No. (%) | 25 (18) | 82 (38) | 2.37 (1.73-3.26) | <0.01* |
| OSI, median (IQR) | 18 (14-22) | 20 (14-25) | 1.04 (1.02-1.06) | <0.01* |
| OSI < 17.0 (mild), No. (%) | 70 (48) | 83 (36) | 1 |  |
| 17.0 < OSI < 23.1 (moderate), No. (%) | 48 (33) | 66 (28) | 1.04 (0.75-1.43) | 0.82 |
| OSI > 23.1 (severe), No. (%) | 28 (19) | 84 (36) | 2.14 (1.58-2.91) | <0.01* |
| Comorbidity |  |  |  |  |
| Hypertension, No. (%) | 77 (53) | 101 (43) | 0.68 (0.52-0.88) | <0.01* |
| Liver cirrhosis, No. (%) | 10 (7) | 34 (15) | 1.67 (1.16-2.4) | <0.01* |
| Malignancy, No. (%) | 20 (14) | 68 (29) | 1.58 (1.19-2.1) | <0.01* |
| Treatment received during ARDS |  |  |  |  |
| Vasopressor, No. (%) | 87 (60) | 207 (89) | 3.09 (2.05-4.66) | <0.01* |
| Systemic steroid, No. (%) | 111 (76) | 209 (90) | 1.72 (1.13-2.63) | 0.01* |
| Prone position, No. (%) | 11 (8) | 31(13) | 1.69 (1.15-2.46) | <0.01* |
| Continuous hemofiltration, No. (%) | 23 (16) | 103 (44) | 1.98 (1.53-2.58) | <0.01* |
| V_T_/PBW (ml/Kg) | 9 (8-10) | 9 (8-10) | 1.00 (0.99-1.00) | 0.01* |
| P_a_O_2_/F_I_O_2_ ratio | 105 (80-142) | 88 (66-120) | 0.94 (0.92-0.96) | 0.04* |
| C_RS_ (ml/cmH_2_O) | 29 (25-34) | 25 (20-29) | 1.03 (1.02-1.04) | <0.01* |
| Plateau Pressure (cmH_2_O) | 31 (28-33) | 33 (31-36) | 1.09 (1.02-1.16) | <0.01* |
| PEEP (cmH_2_O) | 10 (9-12) | 11 (10-12) | 1.07 (1.04-1.1) | 0.01* |
| Driving Pressure^a^ (cmH_2_O) | 20 (17-22) | 22 (20-25) | 1.00 (0.99-1.00) | <0.01* |

^a^Putative numbers, subject to over-estimation. See Method section for details.

*P < 0.05
