# Supplementary figures and images for: Categorizing Acute Respiratory Distress Syndrome with Different Severities by Oxygen Saturation Index
Source: Diagnostics (Basel). 2023 Dec 24;14(1):37. doi: 10.3390/diagnostics14010037 (PMC10795683; doi:10.3390/diagnostics14010037)

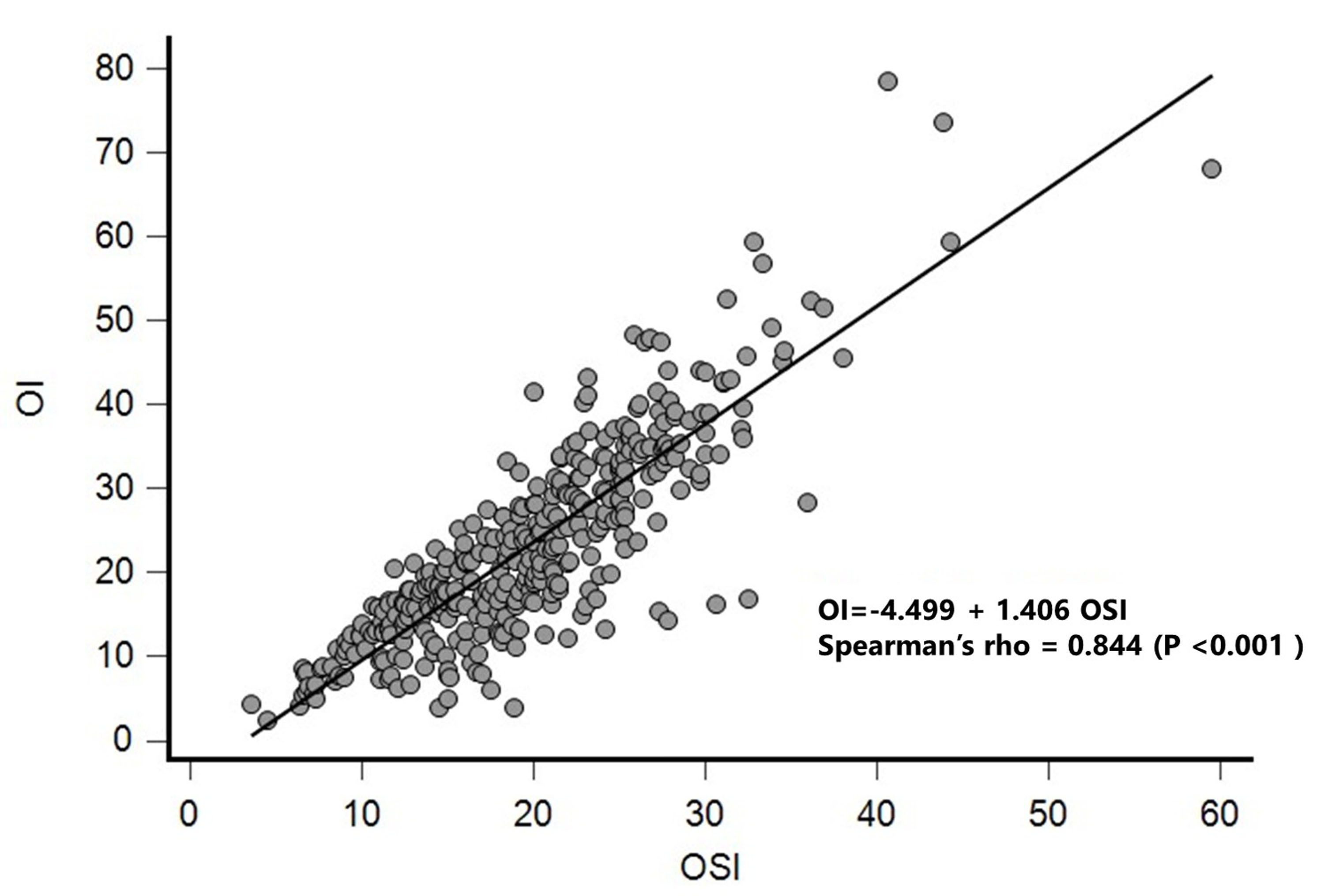

Supplement: Supplementary file 1 [file diagnostics-14-00037-s001.zip › Fig s1.jpg]

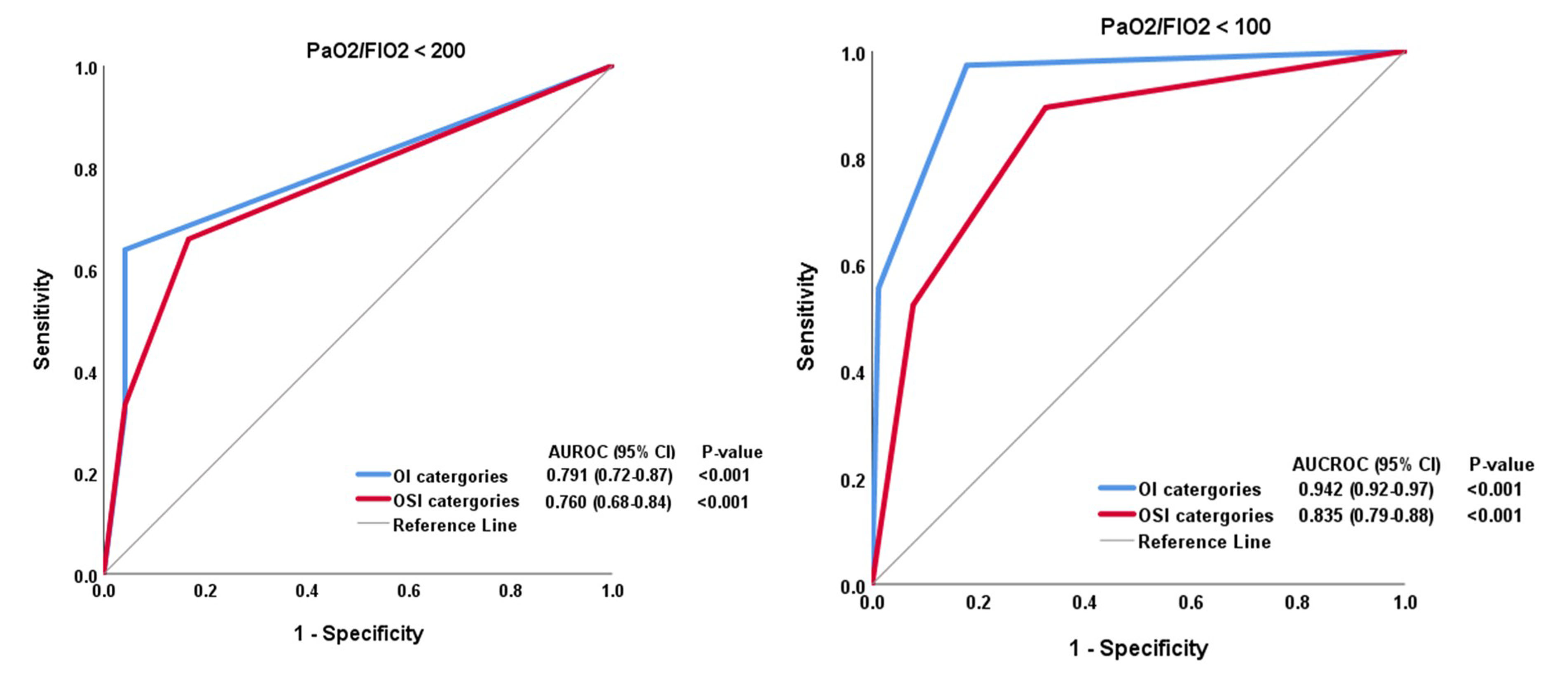

Supplement: Supplementary file 1 [file diagnostics-14-00037-s001.zip › Fig s2.jpg]
